# Distinct cerebral perfusion patterns and linguistic profiles in Alzheimer’s disease-related primary progressive aphasia

**DOI:** 10.1007/s10072-025-08100-2

**Published:** 2025-03-24

**Authors:** Kazuto Katsuse, Kazuo Kakinuma, Nobuko Kawakami, Shoko Ota, Nanayo Ogawa, Ai Kawamura, Chifumi Iseki, Masashi Hamada, Tatsushi Toda, Minoru Matsuda, Shigenori Kanno, Kyoko Suzuki

**Affiliations:** 1 Department of Behavioral Neurology and Cognitive Neuroscience, Tohoku University Graduate School of Medicine, 2-1 Seiryo, Sendai, 980-8575 Japan; 2 Department of Neurology, Graduate School of Medicine, The University of Tokyo, Tokyo, Japan; 3 Department of Neurology, Izumi no Mori Clinic, Yamato, Japan

**Keywords:** Primary progressive aphasia, Alzheimer’s disease, Cerebral perfusion, Single-photon emission computed tomography, Sparse principal component analysis

## Abstract

Alzheimer’s disease (AD)-related primary progressive aphasia (PPA) exhibits considerable heterogeneity in clinical presentation and neuroimaging patterns. No studies have quantitatively assessed cerebral perfusion patterns or systematically evaluated the internal heterogeneity of linguistic and neuroimaging features in this population. This study aimed to investigate cerebral hypoperfusion patterns and elucidate their correlation with diverse linguistic features in patients with AD-related PPA using a data-driven approach. Eleven patients with AD-related PPA and 34 with non-AD-related PPA were categorized based on cerebrospinal fluid biomarkers, and their single-photon emission computed tomography (SPECT) data were analyzed. Cerebral hypoperfusion was assessed across 56 regions of interest (ROIs) covering the entire cerebral hemisphere. Sparse principal component (sPC) analysis was performed on the AD-related PPA group to identify distinct patterns of cerebral perfusion reduction and correlate these components with clinical assessments of linguistic abilities. AD-derived sPCs were identified, reflecting hypoperfusion patterns in the left temporoparietal, frontal, and temporal pole regions, corresponding to regions typically associated with logopenic, nonfluent, and semantic variants. In both AD-PPA and non-AD-PPA, the sPC corresponding to the anterior temporal region was associated with semantic comprehension deficits, whereas that corresponding to the frontal region was linked to nonfluent speech and Kana writing impairment. sPC-based hierarchical clustering revealed clusters corresponding to logopenic, nonfluent, and semantic variants, with the anomic subtype distinguished from logopenic PPA. AD-positive cases were distributed across these clusters, emphasizing AD-PPA heterogeneity. These findings suggested that AD-related PPA heterogeneity is reflected in distinct cerebral perfusion patterns, which correlate with varying linguistic deficits.

**Supplementary Information:**

The online version contains supplementary material available at 10.1007/s10072-025-08100-2.

## Introduction

Primary progressive aphasia (PPA) is a distinct neurodegenerative syndrome characterized by progressive decline in linguistic skills [1, 2]. The current consensus identifies three major PPA variants: nonfluent/agrammatic (nfvPPA), semantic (svPPA), and logopenic (lvPPA). Differentiating among these variants is clinically important, as they are associated with distinct underlying pathologies: svPPA is linked to TDP-43 type C; nfvPPA to tauopathy, Pick’s disease, or TDP-43 type A; and lvPPA to Alzheimer’s disease (AD) or *GRN* mutations [2–6]. Recent research, however, has demonstrated that Alzheimer’s pathology is not limited to lvPPA but also plays a role in nfvPPA, and, although less common, in svPPA [7–9], resulting in a broader spectrum of symptoms. Moreover, 6–41% of PPA cases do not exhibit clear characteristics of one of these variants and are thus categorized as unclassified PPA (ucPPA) [5, 10, 11]. These ucPPA cases include mixed subtypes, which meet the criteria for multiple variants, and anomic subtype, which is characterized by isolated anomia that does not fit the criteria for any specific variant, further complicating classification. Meta-analyses of PPA have indicated that approximately half of ucPPA cases are linked to AD pathology [5, 12], suggesting that some of the variability observed in PPA reflects AD-related heterogeneity [13–15].

To address this, some researchers have proposed moving beyond classical variant categorization and instead focusing on the features of AD pathology in PPA cases, collectively termed “Alzheimer-related aphasia” [13]. To elucidate the heterogeneity of AD pathology in PPA, studies have focused on neuropsychological and neuroimaging features in patients with amyloid biomarkers confirmed through cerebrospinal fluid (CSF) analysis or amyloid-positron emission tomography (PET) [16]. A previous study on PPA with underlying AD pathology found that while most cases were classified as lvPPA, approximately one-third of patients exhibited agrammatism, whereas some presented only anomia with preserved repetition, highlighting the clinical diversity of AD-related PPA (AD-PPA) [17]. Despite this clinical heterogeneity, structural magnetic resonance imaging (MRI) revealed no significant differences in atrophy patterns between the logopenic and agrammatic groups, with approximately half of the cases lacking typical atrophy. Another study focusing on PPA with autopsy-confirmed AD proposed an expanded “logopenic spectrum,” characterized by phonological loop deficits associated with reduced digit span as a core feature [18]. This spectrum includes not only pure lvPPA but also lvPPA−, which is characterized by preserved repetition, and lvPPA+, which presents with additional grammatical or semantic deficits, all of which display common microscopic neurodegenerative pathologies in the superior and middle temporal, angular, and midfrontal cortices. Several studies have examined the logopenic spectrum extensively. One study identified three distinct clusters based on linguistic performance and cortical atrophy patterns: one with pure anomia and left posterior inferior parietal and lateral temporal thinning, a second with mild single-word comprehension deficits and bilateral fusiform thinning, and a third with additional repetition impairments and left superior temporal thinning, highlighting the neuroanatomical and clinical heterogeneity of the logopenic spectrum [19]. Structural MRI analyses of amyloid-PET-positive PPA have revealed cortical thinning not only in the left posterior perisylvian region, which is typical of lvPPA, but also in regions typically affected in nfvPPA (frontal opercular region) and svPPA (ventral temporal region) [20]. A profile analysis based on multidimensional scaling characterized the logopenic and semantic linguistic profiles along a continuum, demonstrating that lvPPA + occupies an intermediate position, with semantic memory impairments associated with altered metabolism in the anterior fusiform gyrus and posterior middle temporal gyrus [21].

These findings suggested that although the widespread cortical involvement in AD-PPA may account for its broader linguistic impairments, a comprehensive framework that reflects individual variability and explains the correspondence between diverse linguistic profiles and the cortical abnormalities observed in neuroimaging studies has yet to be established. Despite the limited studies specifically applied to AD-PPA, many researchers have begun to adopt data-driven approaches, such as principal component analysis (PCA) or hierarchical clustering, to analyze heterogeneous neuropsychological profiles and neuroimaging features [22–26]. These methods aim to classify linguistic and anatomical variability objectively, offering the potential for a more comprehensive understanding of heterogeneity without relying on predefined diagnostic criteria.

One challenge in linking diverse linguistic profiles to neuroimaging features is the limited sensitivity of structural MRI in detecting early-stage atrophy in PPA [17]. Although fluorodeoxyglucose (FDG)-PET studies provide valuable insights, their high cost has led many researchers to continue relying primarily on structural MRI. Brain perfusion single-photon emission computed tomography (SPECT), a cost-effective alternative, may detect changes such as decreased regional cerebral blood flow (rCBF) earlier than MRI-detectable atrophy, facilitating earlier diagnosis of neurodegenerative diseases [27]. SPECT is also useful in diagnosing PPA [1, 28], with studies reporting higher sensitivity than that of MRI and strong correlations between hypoperfusion severity and FDG-PET metabolic patterns [29]. Although comparative studies of amyloid-positive and -negative PPA have revealed a distinct pattern of hypoperfusion extending from the left temporoparietal and dorsolateral prefrontal cortices in AD-PPA cases [30], no studies have quantitatively assessed cerebral perfusion or specifically evaluated the internal heterogeneity of AD-PPA.

In this study, we aimed to analyze cerebral perfusion reduction patterns in AD-PPA using perfusion SPECT through a data-driven analysis. To enable quantitative assessment, we defined a novel measure of perfusion reduction, the degree of cerebral perfusion reduction (DCPR), which considers both the extent and severity of hypoperfusion in each region of interest (ROI). We used this measure to capture individual perfusion reduction patterns, which we then subjected to PCA. To address the challenge posed by a limited number of participants and many variables, we employed a sparse principal component analysis (sPCA), which applies constraints that force many component loadings to be zero, thereby preventing overfitting and enhancing interpretability [31]. We hypothesized that categorizing the neuroimaging features of AD-PPA would uncover distinct patterns associated with linguistic profiles in established neuroanatomical frameworks. By correlating these cerebral perfusion patterns with linguistic characteristics, our objective was to enhance the understanding of the heterogeneity present in AD-PPA and its associated clinical spectrum. We applied the principal components derived from AD-PPA to both AD-PPA and non-AD-PPA cases, using hierarchical clustering based on correlation distance to elucidate AD diversity within the PPA spectrum and distinguish it from non-AD-PPA. This approach aimed to bridge the gap between neuroimaging findings and linguistic outcomes, ultimately contributing to a more comprehensive characterization of AD-PPA.

## Materials and methods

### Participants

This retrospective study included native Japanese speakers who were admitted to the Department of Behavioral Neurology and Cognitive Neuroscience, Tohoku University Hospital for evaluation between April 2018 and January 2024. Inclusion criteria required patients to meet the diagnostic criteria for PPA [2]. Exclusion criteria were as follows: a history of unrelated central nervous system diseases (e.g., stroke, head trauma, epilepsy); concurrent psychiatric disorders; a history of drug/alcohol abuse; and prominent initial episodic memory, visuospatial impairments, or behavioral disturbances. Of the 55 consecutive patients diagnosed with PPA, 45 right-handed individuals who consented to CSF testing for amyloid-β and tau biomarkers were enrolled in this study. All underwent neurological and neuropsychological evaluations by neurologists and speech-language pathologists, in addition to neuroradiological studies including brain MRI and perfusion SPECT. Based on multiple verbal assessments and neuroradiological imaging findings, patients were classified into nfvPPA, svPPA, lvPPA, or ucPPA, according to established criteria [2]. For nfvPPA diagnosis, the presence of apraxia of speech was determined through assessing phonetic and prosodic features in spontaneous speech, based on consensus among multiple experienced neurologists and speech-language pathologists. The presence of agrammatism was assessed through evaluating the misuse or omission of case particles in spontaneous speech or writing, which is considered an agrammatism characteristic in the Japanese language, as determined by consensus among experienced neurologists and speech-language pathologists. The ucPPA category was further divided into mixed subtypes that meet the criteria for multiple variants and anomic subtypes, characterized by isolated anomia that does not fulfill any subtype criteria. The medical records were evaluated to obtain information regarding age, sex, handedness, duration of illness prior to evaluation, history of education, Clinical Dementia Rating (CDR), Movement Disorder Society-Unified Parkinson’s Disease Rating Scale (MDS-UPDRS) part III score, and Mini-Mental Status Examination (MMSE) score.

### Language evaluation

The Japanese version of Western Aphasia Battery (WAB) was used to assess linguistic skills, with the aphasia quotient (AQ) serving as an indicator of overall linguistic abilities. Given that two writing systems are used in the Japanese language, Kanji (morphograms) and Kana (syllabograms), six-word dictation tasks were conducted separately for each script. Additional tasks included the confrontation naming and single-word auditory comprehension of 100 high- and low-familiarity objects, verb naming and comprehension tasks (40 words each) from the Test of Lexical Processing in Aphasia (TLPA), letter fluency, semantic fluency, and digit span forward and backward tasks. Auditory comprehension was further assessed using the shortened Japanese version of the Token Test [32]. Part I–V comprises 23 items that reflect verbal short-term and working memory, while part VI comprises 13 items with more complex instructions, reflecting syntactic comprehension and grammatical deficits.

### CSF study

CSF samples were collected via lumbar puncture, immediately centrifuged, and analyzed at LSI Medience Co., Japan. Levels of amyloid beta 1–42 (Aβ1–42), total tau (t-tau), and phosphorylated tau (p-tau) were measured using enzyme-linked immunosorbent assay kits (Wako Pure Chemical Industries and Nipro Corp., Japan). Given that the ratio of Aβ1–42 to t-tau enhances the sensitivity and specificity for AD diagnosis [33], two biomarkers were used: the Aβ1–42/t-tau ratio and p-tau levels. To assess the heterogeneity within the AD-PPA group, a conservative diagnostic approach was adopted, classifying cases as AD-PPA only when both Aβ1–42/t-tau < 1.895 and p-tau > 60 pg/mL were met. Cases that did not fulfill either criterion were classified as non-AD-PPA.

### Perfusion SPECT analysis

All patients underwent ^123^I-iodoamphetamine (^123^I-IMP)-SPECT within 60 d of the neurological and neuropsychological assessments. SPECT images were collected using a dual-detector variable angle gamma camera (Symbia E; Siemens Healthcare, Erlangen, Germany) equipped with a parallel hole low-medium-energy general purpose collimator. Projection data were acquired for approximately 15 min after an intravenous injection of 111 MBq of N-isopropyl-4-p-[^123^I]-iodoamphetamine (Nihon Medi-Physics Co., Japan). The image acquisition time (circular orbit and continuous mode, 150 s/rotation × 12 rotations, 128 × 128 matrix) was 30 min. The energy peak was set at 159 keV with a 20% energy window. Reconstructed transaxial images (slice thickness: 3.3 mm) used a Butterworth filter (cutoff frequency: 0.37 cycles/cm, order: 8), with attenuation correction applied using the Chang’s method.

The SPECT control group included 29 cognitively healthy individuals (mean age: 64.2 ± 8.2 years; 19 women, 10 men) who met the following criteria: (1) aged 50–80 years at the time of informed consent, (2) MMSE score > 27 within 3 months of imaging, and (3) no MRI or MRA abnormalities within 3 months of imaging. Pixel-based comparisons of rCBF between patients with AD-PPA or non-AD-PPA and the normal control cohort were conducted using three-dimensional stereotactic surface projections (3D-SSP) [34]. Anatomical standardization was performed to align the brain of each patient with the Talairach coordinate system, with cortical count data being projected onto the brain surface. For each pixel on the brain surface, the mean and standard deviation of the normal control group (normalized to the cerebellum) were compared with the values of each individual patient to calculate *Z*-scores, generating a *Z*-map for individual analysis.

Pixel values from the 3D-SSP data were aggregated for each ROI using the stereotactic extraction estimation method [35] (medi + FALCON^®^, Nihon Medi-Physics Co.). For regions outside the lateral temporal lobe, level three lobule-based ROIs were employed, resulting in seven ROIs in each frontal lobe, six ROIs in each parietal lobe, four ROIs in each occipital lobe, and three ROIs in both the basal and medial regions for each hemisphere (Supplementary Table 3). In contrast, for the lateral temporal lobe, lobule-based ROIs include four ROIs from the superior, middle, inferior, and transverse temporal gyri. However, considering the anterior temporal lobe’s role in semantic processing in PPA, level 5 Brodmann area (BA) ROIs (BA 22, 21, 20, 38, 42) were selected to capture this critical region. In total, 56 ROIs (28 per hemisphere) were defined. For each ROI, the percentage of pixels with a *Z*-score > 2 and the mean *Z*-score were calculated. Their product was used to quantify the DCPR in each ROI, with positive values indicating reduced cerebral perfusion.

### sPCA

The sPCA approach was applied to the DCPR patterns in the AD-PPA group across 56 ROIs to characterize cerebral hypoperfusion patterns and assess AD-PPA heterogeneity. sPCA was chosen instead of standard PCA because of the small number of participants and for facilitating data interpretation. For sPCA, the perfusion patterns of four consecutive patients with biomarker-confirmed Alzheimer’s type dementia without linguistic symptoms were included, all of whom were hospitalized during the same period. sPCA was performed using the SparsePCA module from scikit-learn library (v1.1.3) with default parameters (alpha = 1, ridge_alpha = 0.01, and max_iter = 1000), yielding five sparse principal components (sPCs) that explained the greatest variance. Individual DCPR data were then projected onto these sPCs using a least squares approach. To examine the relationship between imaging patterns and clinical profiles, Spearman’s correlations coefficients were calculated between sPCs and linguistic features in the AD-PPA group. In addition, the sPCs identified in the AD-PPA group were applied to the non-AD-PPA group, and correlations with linguistic features were analyzed to explore cross-group patterns.

### Statistical analysis and hierarchical clustering

Statistical analyses were conducted using Python (v3.10.6) with relevant libraries, including SciPy (v1.9.3) and pandas (v1.5.1). Group comparisons were performed using the Wilcoxon rank-sum test, whereas associations between categorical variables were assessed using the chi-square test. The Spearman’s rank correlation coefficient was used to evaluate correlations between variables. For all AD-PPA and non-AD-PPA cases, hierarchical clustering was performed using the Ward’s method based on the correlation distance of the obtained sPCs, and implemented using the scipy.cluster.hierarchy module.

## Results

### Demographics

The AD-PPA group comprised 11 patients (#P1–#P11) meeting the criteria of Aβ1–42/t-tau < 1.895 and p-tau > 60 pg/mL, whereas the non-AD-PPA group included 34 patients (#NP1–#NP34). In the non-AD-PPA group, three patients met only one of the two criteria: #NP34 satisfied Aβ1–42/t-tau < 1.895 only, whereas #NP4 and #NP7 met p-tau > 60 pg/mL only. Patient demographics are summarized in Table 1. In the AD-PPA group, the clinical subtypes included 4 cases of lvPPA (#P1–#P4), 1 case of nfvPPA (#P5), 1 case of svPPA (#P6), and 5 cases of ucPPA (of which four were anomic [#P7–#P10] and one was mixed nf + lv [#P11]). In contrast, the non-AD-PPA group comprised 25 cases of nfvPPA (#NP1–#NP25), 6 cases of svPPA (#NP26–#NP31), and 3 cases of ucPPA (#NP32–#NP34); notably, no lvPPA cases were present. The results of the neuropsychological evaluations for each of the 11 patients with AD-PPA and the 34 patients with non-AD-PPA are detailed in Supplementary Tables 1 and 2, respectively. We observed that MMSE scores were significantly lower in the AD-PPA group. Linguistically, we did not detect any significant differences in WAB AQ or digit span task performance between the groups. In addition, we found that in the AD-PPA group, apraxia of speech and agrammatism were less prevalent, while object naming and Kanji word dictation performance were significantly lower than those in the non-AD-PPA group.

**Table 1:** Demographics of AD-PPA group and non-AD-PPA group

	AD-PPA (*n* = 11)	non-AD-PPA (*n* = 34)
Age at exam [years]	74.2 (6.88)	71.3 (7.13)
Disease duration [years]	3.68 (2.55)	2.75 (1.66)
Sex [female/male]	6/5	12/22
Clinical subtype	lv 4; nfv 1; sv 1; uc 5	nfv 25; sv 6; uc 3
CSF		
p-tau [pg/mL]	96.1 (39.1)*	38.4 (12.0)
Aβ1–42/t-tau	1.08 (0.46)*	5.39 (1.69)
Education [years]	13.5 (2.34)	13.0 (2.70)
CDR, overall	0.727 (0.467)	0.500 (0.369)
MDS-UPDRS III (/132)	6.00 (6.59)	10.0 (13.9)
MMSE (/30)	18.5 (4.89)*	22.7 (5.86)
RCPM (/36)	24.3 (4.80)	26.6 (6.23)
Apraxia of speech (%)	18.2%*	73.5%
Agrammatism (%)	9.1%*	67.6%
WAB		
Aphasia Quotient (/100)	78.1 (14.9)	74.2 (15.6)
Spontaneous speech (/20)	15.0 (2.61)	13.6 (3.92)
Auditory comprehension (/10)	8.62 (1.52)	8.26 (1.48)
Repetition (/10)	8.21 (1.89)	8.40 (2.14)
Naming (/10)	6.11 (2.29)	6.87 (2.44)
Reading (/10)	8.02 (1.54)	8.20 (1.86)
Writing (/10)	7.27 (1.89)	8.14 (1.88)
Kanji word dictation (/6)	2.82 (1.75)*	4.39 (1.52)
Kana word dictation (/6)	5.55 (0.76)	5.47 (1.42)
Praxis, left (/10)	9.48 (0.66)	8.95 (1.19)
Praxis, right (/10)	9.45 (0.68)	8.97 (1.23)
Constructional/visuospatial task (/10)	7.75 (1.17)	8.11 (1.46)
Calculation (/24)	20.4 (5.71)	21.8 (4.76)
Token test		
Part I-V (/23)	20.1 (3.51)	19.5 (3.65)
Part VI (/13)	7.09 (2.74)	6.33 (4.05)
TLPA		
Naming, high-familiarity objects (/100)	65.9 (16.9)*	78.5 (20.8)
Naming, low-familiarity objects (/100)	30.6 (20.0)*	52.5 (26.0)
Auditory comprehension, high-familiarity objects (/100)	90.0 (6.07)	88.4 (20.0)
Auditory comprehension, low-familiarity objects (/100)	81.5 (9.88)	81.4 (24.9)
Naming, verb (/40)	23.5 (10.3)	28.6 (8.69)
Auditory comprehension, verb (/40)	37.2 (2.64)	36.0 (6.65)
Span		
Digit, forward	4.64 (1.12)	4.46 (1.06)
Digit, backward	3.18 (0.75)	3.53 (1.08)
Letter fluency (ka)	4.27 (2.83)	4.13 (3.71)
Semantic fluency (animal)	6.73 (3.93)	7.53 (4.32)

* Statistically significant (*p* < 0.05)

### Perfusion SPECT analysis

**Fig. 1:**
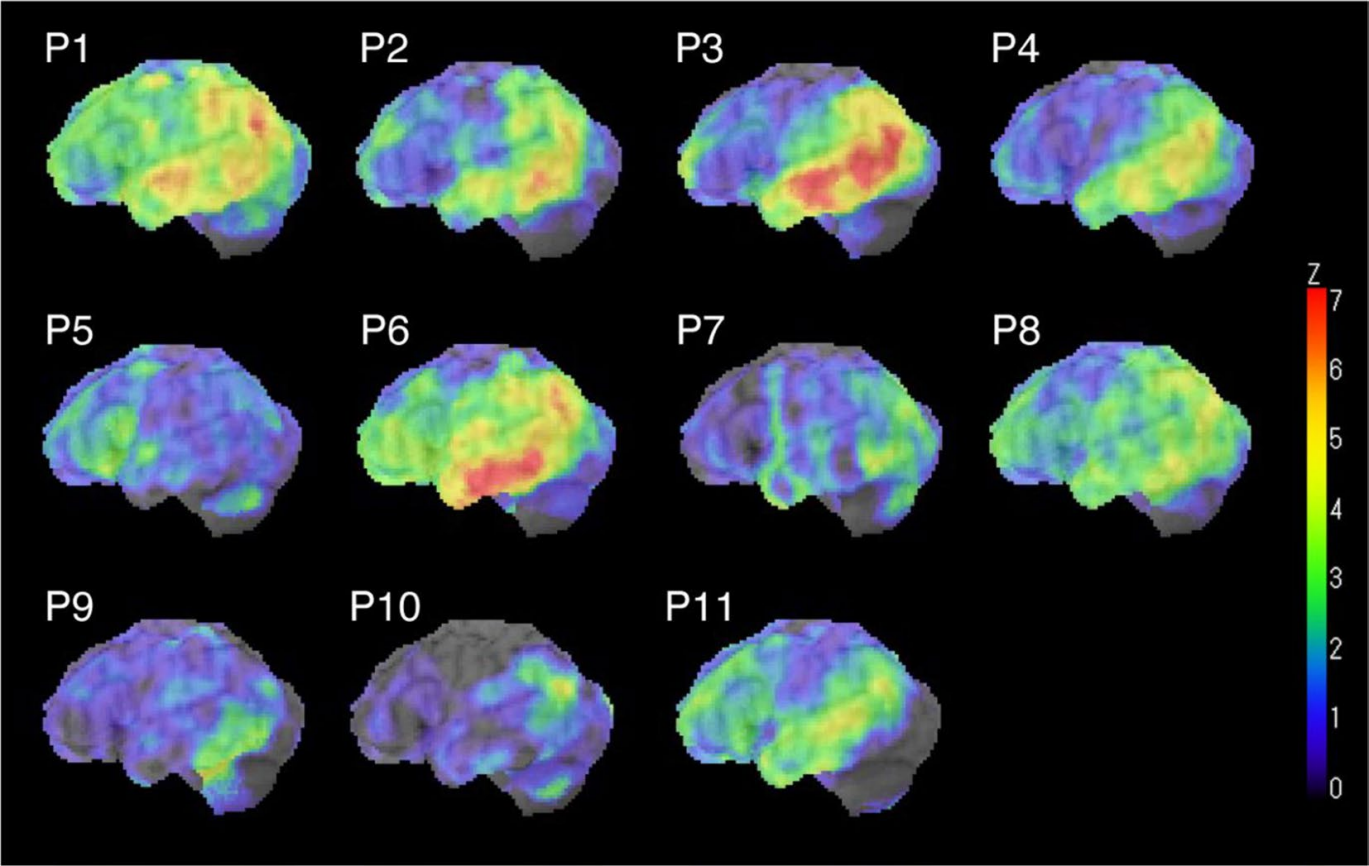
*Z*-map of hypoperfusion for the left hemisphere in each patient with AD-PPA compared with that from the healthy control group

Figure 1 presents a *Z*-map of perfusion SPECT data for the left hemisphere in each patient with AD-PPA, compared with that from the healthy control group. The lvPPA cases (#P1–#P4) consistently exhibited perfusion reductions in the left temporoparietal region. However, we found that one nfvPPA case (#P5) exhibited reduced perfusion in the left frontal lobe, whereas one svPPA case (#P6) showed decreased perfusion in the left inferior temporal lobe. We detected mild perfusion reductions in the anomic PPA cases, which were relatively localized to the left parietal and temporal lobes, except for #P8. The mixed PPA case (#P11) also demonstrated decreased perfusion in the left temporoparietal region. *Z*-maps for the patients with non-AD-PPA compared with those of the healthy control group, are shown in Supplementary Fig. 1.

**Fig. 2:**
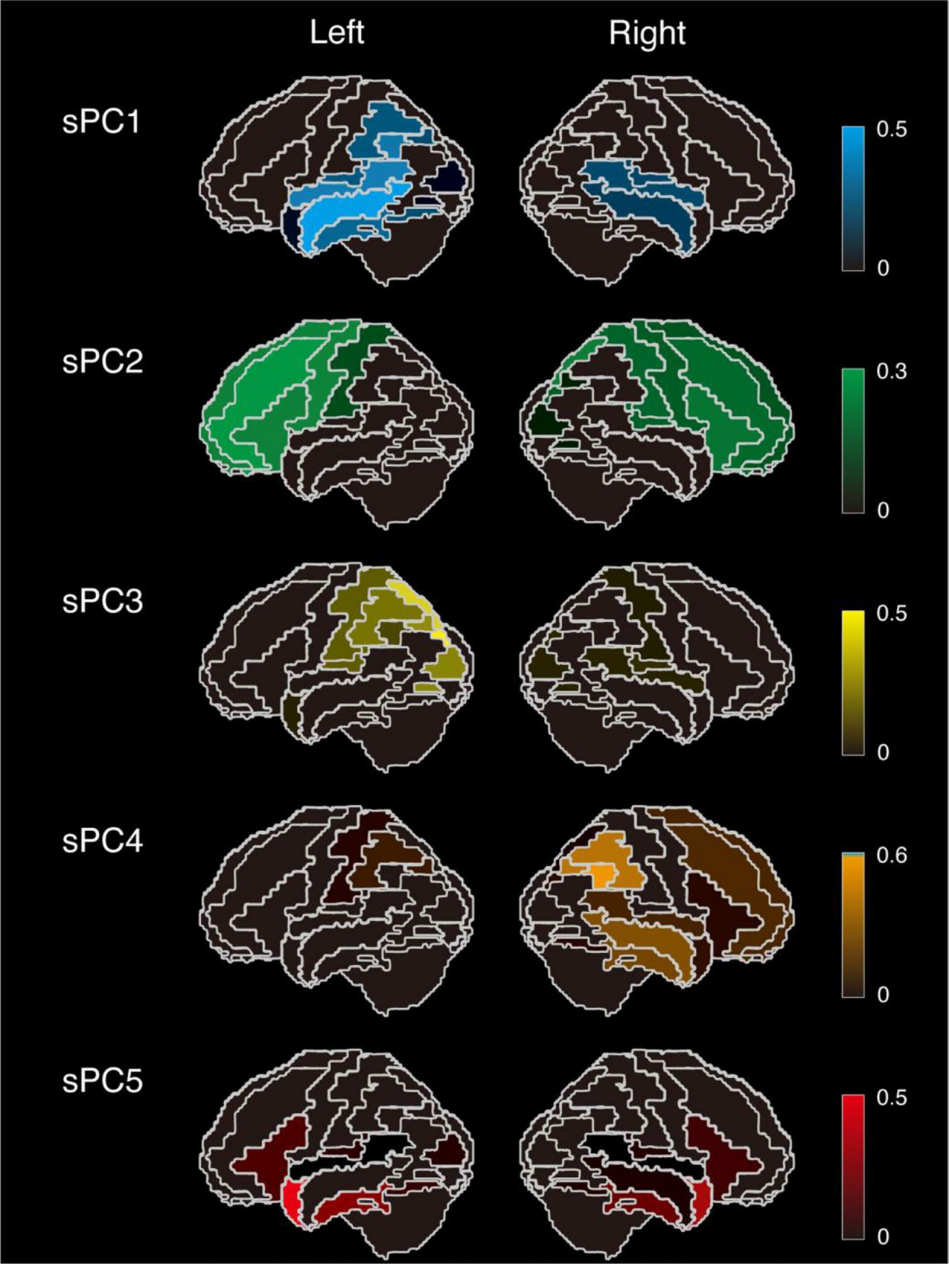
Each sparse principal component (sPC) is displayed on the cortical surface with color coding. sPC1 shows non-zero components predominantly in the bilateral temporoparietal regions with left dominance; sPC2 in the bilateral frontal lobes with left dominance; sPC3 in the left parieto-occipital region; sPC4 in the right frontal, temporal, and parietal lobes; and sPC5 in the bilateral temporal poles and inferior temporal gyri with left dominance

### sPCA results

The results of applying sPCA to the DCPR obtained from the SPECT analysis of AD-PPA cases are summarized in Supplementary Tables 3 and 4 for the left and right hemisphere ROIs, respectively. Figure 2 displays the values of each sPCs on the cortical surface. We found that sPC1 exhibited its highest contributions in the left superior and middle temporal regions, extending to the left supramarginal, transverse temporal, and inferior temporal areas. sPC2 demonstrated widespread contributions across the left frontal lobe. sPC3 exhibited its highest values in the left superior parietal and superior occipital regions, along with contributions in the left parieto-occipital area. sPC5 displayed left-sided dominance, while also being significantly involved in the bilateral temporal poles and parahippocampal regions. In contrast, sPC4 was primarily localized to the right parietal lobe, while exhibiting certain contributions in the right temporal and frontal lobes. The DCPR data for each participant, projected onto these sparse components using a least squares approach, are summarized in Table 2. The explained variance for each component was as follows: sPC1 accounted for 20.5%, sPC2 for 15.0%, sPC3 for 13.2%, sPC4 for 10.8%, and sPC5 for 9.3% of the total variance. Owing to sPC4 showing values close to zero in the left hemisphere, whereas non-zero values in the right hemisphere, we restricted all subsequent correlation analyses using linguistic features to sPC1, sPC2, sPC3, and sPC5.

**Fig. 3:**
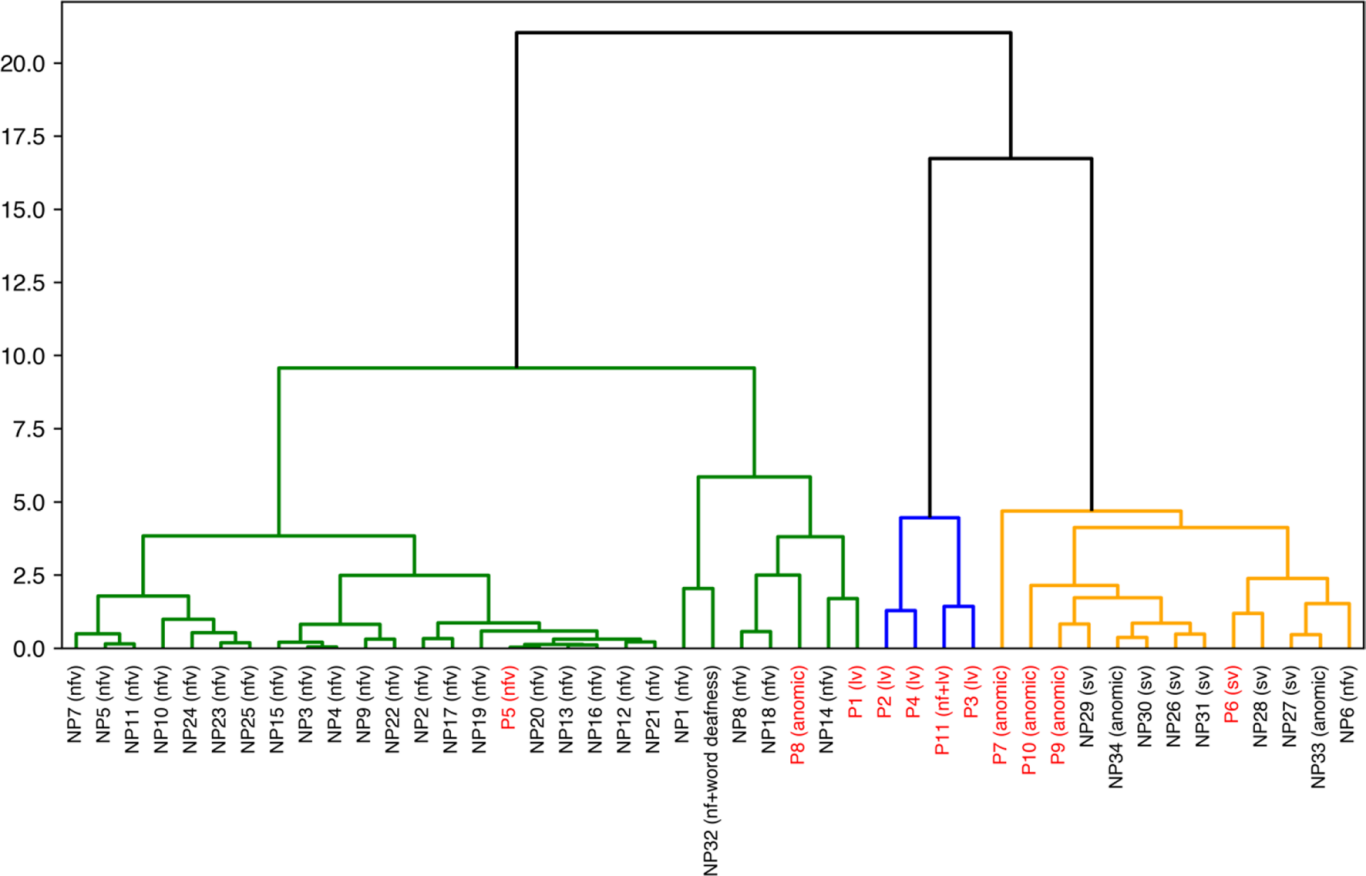
Hierarchical clustering using correlation distance based on the components of sPC. The vertical axis represents the squared Euclidean distance. The clustering resulted in three major clusters: the left cluster (green) primarily consisted of nfvPPA, the middle cluster (blue) included lvPPA and mixed PPA, and the right cluster (orange) mainly comprised svPPA and anomic PPA. AD-PPA cases are highlighted in red

**Table 2:** SPECT perfusion patterns projected onto sPCs for each participant in AD-PPA group

Patient	Subtype	sPC1	sPC2	sPC3	sPC4	sPC5
**P1**	lv	4.314	5.199	4.390	4.378	3.089
**P2**	lv	2.668	-1.044	-1.175	0.870	0.032
**P3**	lv	6.098	-1.852	4.065	-1.284	-0.013
**P4**	lv	2.365	-2.624	-1.084	-0.895	-0.051
**P5**	nfv	-4.354	-0.694	-3.557	-3.856	-1.910
**P6**	sv	6.438	3.620	0.711	2.891	7.686
**P7**	anomic	-3.031	-2.959	0.410	-0.778	1.804
**P8**	anomic	1.224	3.373	3.417	0.949	2.232
**P9**	anomic	-3.660	-2.590	-3.373	-3.614	-1.173
**P10**	anomic	-3.421	-2.655	-2.650	-2.670	-1.871
**P11**	nf + lv	2.495	-1.416	3.596	1.613	-1.501

When we applied the AD-PPA-derived sPCs to the DCPR of non-AD-PPA, the explained variance was 14.8% for sPC2, 12.1% for sPC5, 11.3% for sPC1, 6.8% for sPC4, and 4.7% for sPC3. This distribution likely reflected the higher proportion of nfvPPA and svPPA cases in the non-AD-PPA group, leading to greater variance in the frontal and anterior temporal regions.

The graphs of sPC values for all cases in both groups, with markers distinguishing clinical subtypes, are shown in Supplementary Fig. 2.

### Association of sPCs with linguistic features

The Spearman’s correlations between the sPCs and linguistic features of the AD-PPA group are presented in Table 3 (left column). sPC1, reflecting the left temporoparietal hypoperfusion, correlated strongly with AQ, naming, and repetition tasks in the WAB. It was also strongly correlated with auditory comprehension and reading in the WAB, Token test Part I–V, naming of objects and verbs, auditory comprehension of verbs in the TLPA, and the semantic fluency. sPC2 correlated with writing in the WAB, particularly Kana word dictation, verb naming, and semantic fluency. sPC3 was associated with repetition, verb naming, and auditory comprehension of verbs in the TLPA, while sPC5 correlated with the auditory comprehension of high-familiarity objects, and the naming and auditory comprehension of verbs in the TLPA. We did not detect any significant correlations with spontaneous speech in the WAB, digit span, or letter fluency.

**Table 3:** Spearman correlations between sPCs and linguistic features in AD-PPA group and non-AD-PPA group

	Spearman ρ							
Tests	AD-PPA group				non-AD-PPA group			
	sPC1	sPC2	sPC3	sPC5	sPC1	sPC2	sPC3	sPC5
WAB								
Aphasia Quotient	-0.755**	-0.273	-0.473	-0.155	-0.128	-0.309**†**	-0.014	-0.178
Spontaneous speech	-0.464	-0.520	-0.218	0.005	0.047	-0.221	0.044	0.069
Auditory comprehension	-0.724*	-0.237	-0.346	-0.096	-0.086	-0.315**†**	-0.035	-0.126
Repetition	-0.685*	-0.210	**-0.562†**	-0.096	-0.150	-0.414*	-0.075	0.000
Naming	-0.773**	-0.118	-0.373	-0.427	-0.198	-0.083	-0.014	-0.516**
Reading	-0.603*	-0.443	-0.292	-0.192	-0.125	-0.118	0.062	-0.172
Writing	**-0.547†**	-0.702*	-0.296	-0.433	-0.029	-0.297**†**	-0.082	-0.085
Kanji word dictation	-0.425	-0.489	-0.114	-0.283	-0.104	-0.158	-0.107	-0.244
Kana word dictation	-0.175	**-0.546†**	0.220	-0.130	-0.293	-0.620**	-0.440*	-0.369*
Praxis, left	-0.268	0.117	0.207	0.075	-0.072	-0.167	-0.025	-0.290**†**
Praxis, right	-0.252	0.168	0.201	0.093	0.036	-0.101	0.010	-0.221
Constructional/visuospatial	-0.305	-0.428	-0.196	-0.082	0.195	-0.308**†**	-0.140	0.118
Calculation	**-0.568†**	-0.405	-0.337	-0.472	0.252	-0.183	0.026	0.051
Token test								
Part I-V	-0.604*	0.189	-0.401	-0.134	0.019	-0.353**†**	-0.333**†**	0.189
Part VI	-0.256	0.037	0.028	0.191	-0.150	-0.490**	-0.355**†**	0.011
TLPA								
Naming, high-familiarity objects	-0.645*	-0.200	-0.355	-0.391	-0.277	-0.119	0.003	-0.549**
Naming, low-familiarity objects	-0.788**	-0.141	-0.401	-0.451	-0.402*	-0.012	0.002	-0.682**
Auditory comprehension, high-familiarity objects	-0.475	-0.313	-0.313	**-0.548†**	-0.195	-0.053	-0.103	-0.312**†**
Auditory comprehension, low-familiarity objects	-0.638*	-0.118	-0.492	-0.360	-0.187	-0.077	-0.005	-0.414*
Naming, verb	-0.866**	-0.661*	-0.711*	**-0.528†**	-0.232	-0.122	-0.021	-0.367*
Auditory comprehension, verb	-0.902**	-0.248	**-0.594†**	**-0.524†**	-0.173	-0.027	-0.062	-0.256
Span								
Digit span, forward	-0.183	-0.052	-0.164	0.244	0.095	-0.233	-0.206	0.421*
Digit span, backward	-0.368	-0.432	-0.123	0.069	0.167	-0.029	0.054	0.305**†**
Letter fluency (ka)	-0.297	-0.269	-0.201	-0.023	0.067	-0.270	-0.066	0.187
Semantic fluency (animal)	**-0.593†**	-0.635*	-0.225	-0.280	-0.107	-0.135	-0.052	-0.267

Statistical significance was determined as follows: ^†^*p* < 0.1, ^*^p < 0.05, ^**^p < 0.01

Values in **bold** indicate a statistically significant and moderate or stronger negative correlation (ρ < -0.4)

In the non-AD-PPA group (Table 3, right column), sPC1 showed only a moderate correlation with naming of low-familiarity objects. sPC2 correlated significantly with repetition, Kana word dictation, and Token test Part VI. sPC3 showed a moderate correlation with Kana word dictation, while sPC5 showed significant correlations with naming in the WAB, object naming, and auditory comprehension of low-familiarity objects.

### Hierarchical clustering

Figure 3 presents the results of hierarchical clustering based on correlation distance of the sPCs applied to all PPA cases. Even when using perfusion SPECT data alone, the clustering formed three branches that partially aligned with clinical subtypes: the left branch primarily included nfvPPA, the middle branch was predominated by lvPPA and mixed subtypes, whereas the right branch mainly included svPPA and anomic PPA. Notably, despite being predominantly clustered in the middle and right branches, AD-PPA cases (highlighted in red) were dispersed across all three branches, highlighting the heterogeneity of AD-PPA.

## Discussion

In this study, we investigated the SPECT patterns of cerebral hypoperfusion in patients with AD-PPA using a data-driven sPCA approach and examined the relationship between sPCs and linguistic features in the AD-PPA and non-AD-PPA groups. We initially hypothesized that AD-PPA neuroimaging feature categorization would reveal distinct patterns associated with specific clinical symptoms, thereby clarifying AD-PPA heterogeneity. Our findings confirmed that, even within the AD-PPA group, distinct neuroimaging patterns corresponding to four sPCs (sPC1–3, 5) aligned with different linguistic profiles, enhancing our understanding of AD-PPA variability. When these sPCs were applied to non-AD-PPA, correlations between sPCs and linguistic profiles revealed both similarities and differences between the two groups. Furthermore, hierarchical clustering based on data-driven sPCs from perfusion data largely identified clusters corresponding to the three existing types (nfvPPA, lvPPA, and svPPA), with AD-positive cases being dispersed across these clusters, highlighting AD-PPA heterogeneity and offering a framework for organizing the diverse spectrum of symptoms.

Unlike previous studies that primarily relied on MRI, we developed a novel index, the DCPR, which accounts for both the proportion of affected area and the severity of perfusion deficits within each ROI, based on perfusion SPECT data. Compared with functional imaging techniques, MRI has limitations in detecting early pathological changes. In contrast, ^123^I-IMP-SPECT is more cost-effective and clinically accessible than FDG-PET, while still capturing early perfusion changes and showing a strong correlation with hypometabolic regions. As shown in the *Z*-maps, all patients in our study exhibited detectable perfusion deficits. Defining DCPR across multiple ROIs covering the entire brain enabled the correlation of quantitative analysis with clinical symptoms. In addition, the data-driven approach reduced the complexity of perfusion patterns into interpretable components, while the sparse method, characterized by numerous zero components, facilitated easier interpretation.

The sPCs derived from perfusion data covered a wide range of brain regions, including the temporoparietal (sPC1), frontal (sPC2), parieto-occipital areas (sPC3), and temporal poles/inferior temporal region (sPC5). Notably, the sPC1, sPC2, and sPC5 resembled the patterns seen in lvPPA, nfvPPA, and svPPA, respectively. Given that these sPC patterns were derived exclusively from the AD group, their correlations with distinct linguistic deficits suggested that this neuroimaging variability reflected the clinical AD-PPA heterogeneity. In our cohort, 11 patients were classified as biomarker-confirmed AD-PPA, displaying diverse clinical subtypes. All lvPPA cases were classified as AD-PPA, consistent with previous findings of a high AD prevalence in lvPPA. One case each of nfvPPA and svPPA were identified. However, the most common subtype was ucPPA, with four cases presenting isolated anomia and one exhibiting a mixed subtype. These isolated anomia cases likely corresponded to PPA-L without repetition deficits [17] or lvPPA – [18], highlighting AD-PPA heterogeneity [13, 15, 17, 18, 36]. Contrary to previous findings, which identified impaired digit span as a characteristic of AD-PPA, only 45% of patients with AD-PPA had a forward digit span of < 5, and only 18% had a backward digit span of < 3. No significant differences in digit span were observed between the AD-PPA and non-AD-PPA groups. The six patients (#P1, #P6–#P10) with preserved digit span had an average disease duration of 4.4 years, indicating that these were not simply early-stage cases. These findings suggested that our cohort may display greater variability than previously reported.

Regarding the characteristics of the identified sPCs, sPC1, which accounted for the maximum variance in the AD-PPA group, displayed non-zero components in the left perisylvian temporoparietal region. This pattern closely resembled the hypoperfusion observed in lvPPA and aligned with the most common hypoperfusion patterns observed in AD-PPA. The sPC1 values were high in the four lvPPA cases (#P1–#P4), with one mixed case (#P11) with logopenic features also showing a high sPC1 value. This component correlated strongly with the aphasia severity, repetition, naming, and auditory comprehension in the WAB, Token test Part I–V, and naming of objects and verbs, aligning with the core lvPPA symptoms of word-finding difficulties and verbal short-term memory impairments. In contrast, non-AD-PPA cases showed low variance explained by sPC1, with only a moderate correlation observed with object naming in TLPA, suggesting that the non-AD-PPA group, primarily composed of nfvPPA and svPPA, exhibited minimal perfusion in the parietal-temporal regions.

Although the sPCA focused solely on the AD group, sPC5 was extracted, exhibiting non-zero components in the left-dominant bilateral temporal pole and inferior temporal regions, areas typically impaired in svPPA. Among our patients, one individual with svPPA (#P6) demonstrated a high positive sPC5 value. These left anterior and inferior temporal regions are hypothesized to be crucial for semantic comprehension, and our analysis consistently showed that sPC5 correlated with the auditory comprehension of high-familiarity objects in AD-PPA. When applied to the non-AD-PPA group, this trend was more pronounced, with sPC5 being relatively high in all six svPPA cases (#NP26–#NP31) and correlating with the naming and auditory comprehension of objects. Notably, when comparing the AD-positive svPPA case (#P6) with the AD-negative svPPA cases (#NP26–#NP31), both sPC5 and sPC1 were elevated in #P6 (Supplementary Fig. 2), despite the absence of logopenic features. In AD-PPA, svPPA may exhibit hypoperfusion patterns in both the left perisylvian temporal-parietal and anterior inferior temporal regions. Consistently, recent hierarchical cluster analyses identified a subgroup resembling svPPA, with FDG-PET findings revealing the involvement of both the left anterior temporal and temporoparietal regions, and being associated with AD pathology [22, 26]. In contrast, recent studies have identified a subgroup of patients with AD-PPA with semantic impairment but acceptable repetition, termed a “transcortical sensory aphasia-like pattern” distinct from svPPA [36]. Although not observed in our cohort, a distinct perfusion pattern sparing the Sylvian fissure, crucial for repetition, may emerge in cohorts that include this subtype. Another noteworthy aspect was that the patient with svPPA and AD pathology in our analysis was older (80 years) than those with non-AD pathology (the age range of #NP26–#NP31 was 48–78 years). Previous studies have shown that advanced age is associated with AD-resembling clinical features [37] and higher AD pathology prevalence rather than the typical TDP-43 type C pathology [5]. Based on our findings in conjunction with those of previous studies, svPPA with AD pathology may be associated with relatively advanced age and exhibit reduced perfusion in both the anterior temporal lobe (sPC5) and posterior temporo-parietal regions (sPC1), both of which are associated with semantic comprehension deficits.

The sPC2 exhibited non-zero components in the left-dominant bilateral frontal cortices, which are regions associated with hypoperfusion in nfvPPA. In the AD-positive nfvPPA case (#P5), sPC2 exhibited larger values than sPC1, 3, 4, and 5. Compared with the other components, sPC2 was also relatively high in most nfvPPA cases in the non-AD-PPA group. A significant correlation with handwriting was identified in both groups. Notably, sPC2 correlated significantly with Kana but not with Kanji word dictation, suggesting that sPC2 may reflect Kana writing impairments as left frontal regions are implicated in Kana agraphia. Moreover, in the non-AD-PPA group, sPC2 correlated with Token test Part VI, potentially reflecting syntactic comprehension or agrammatism that are commonly observed in nfvPPA.

These findings suggested that, except for sPC1, the correlations between sPCs and linguistic profiles observed in the non-AD-PPA group are similarly present in the AD-PPA group. In contrast, the non-AD-PPA group exhibited low sPC1 variance, with no strong correlation to linguistic profiles. This difference indicated the heterogeneity in linguistic profiles and hypoperfusion patterns observed in the AD-PPA group. Furthermore, the sPC-based hierarchical clustering effectively distinguished not only AD-PPA cases but also classified non-AD-PPA cases into three major branches corresponding to clinical subtypes: nfvPPA, lvPPA, and svPPA. Among AD-PPA cases, those with anomic PPA (#P7, #P9, and #P10) were predominantly clustered within the svPPA-associated branch, with only one anomic PPA (#P5) in the nfvPPA-associated and none in the lvPPA-associated branch. This suggested that, despite a shared underlying AD pathology, anomic PPA and lvPPA represent distinct entities. The distribution of AD-PPA cases across all three branches further emphasized AD-PPA heterogeneity.

This study had some limitations. First, this was a cross-sectional study, which may reflect the heterogeneity across different disease stages. Recent studies have suggested that the anomic subtype may precede lvPPA or transcortical sensory aphasia-like patterns, which may later progress to Wernicke-like patterns [36]. Moreover, some cases of the anomic subtype exhibit prolonged, localized hypoperfusion patterns alongside language symptoms [38], indicating that variations in disease duration could be significant for pattern classification. Longitudinal studies examining changes in cerebral perfusion over time relative to the progression of linguistic deficits could provide deeper insights into AD-PPA heterogeneity. Second, the potential presence of comorbidities poses another limitation. While the AD group was identified using CSF biomarkers, the potential coexistence of other pathologies, such as tauopathy or TDP-43 [39], which may influence linguistic features, cannot be ruled out. Combining Aβ1–42/Aβ1–40 in the CSF with neuroimaging techniques such as amyloid- and tau-PET would enable more precise evaluations based on the ATN system [16]. Recent autopsy studies have reported cases of late-onset semantic dementia with coexisting AD and TDP-43 type A pathologies [40]. The absence of reliable antemortem biomarkers for TDP-43 highlights the importance of postmortem studies for drawing definitive conclusions about comorbidities and their role in language deficits. Finally, even when similar hypoperfusion patterns are observed, the underlying pathology can lead to different clinical symptoms. For instance, symptomatic differences have been reported between patients with AD-associated and TDP-43-associated semantic dementia [40]. This suggests that that symptom variability, which cannot be fully explained by perfusion patterns alone, may be attributed to disruptions in white matter tracts or broader network-level impairments.

## Conclusion

This study used a data-driven sPCA approach to explore SPECT patterns of cerebral perfusion reduction in patients with AD-PPA. Our findings highlighted the neuroimaging and clinical heterogeneity within the AD-PPA cohort, revealing distinct perfusion patterns associated with various linguistic features. Our results further demonstrated that different sPCs correspond to specific clinical symptoms, enhancing our understanding of the complexities inherent to AD-PPA subtypes. By applying the identified sPCs to the non-AD-PPA group, we revealed both shared and distinct correlations between sPC patterns and linguistic profiles in AD and non-AD groups. The sPC-based hierarchical clustering revealed distinct clusters that aligned with logopenic, nonfluent, and semantic variants, differentiating the anomic subtype from logopenic PPA. Furthermore, AD-positive cases were distributed across these clusters, highlighting AD-PPA heterogeneity. Future longitudinal studies are warranted to further elucidate the progression of cerebral perfusion patterns and their relationship with progressive linguistic deficits over time, thus contributing to a comprehensive framework for understanding AD-PPA diversity.

## Electronic supplementary material

Below is the link to the electronic supplementary material.

Supplementary Material 1

Supplementary Material 2

## Data Availability

Anonymized data supporting the findings of this study will be shared upon request by any qualified investigator.
