# Supplementary material for: Distinct cerebral perfusion patterns and linguistic profiles in Alzheimer’s disease-related primary progressive aphasia
Source: Neurol Sci. 2025 Mar 24;46(7):3071–83. doi: 10.1007/s10072-025-08100-2 (PMC12152036; doi:10.1007/s10072-025-08100-2)

Supplementary Fig. 1. *Z*-map of hypoperfusion for the left hemisphere in each patient with non-AD-PPA compared with that from the healthy control group.

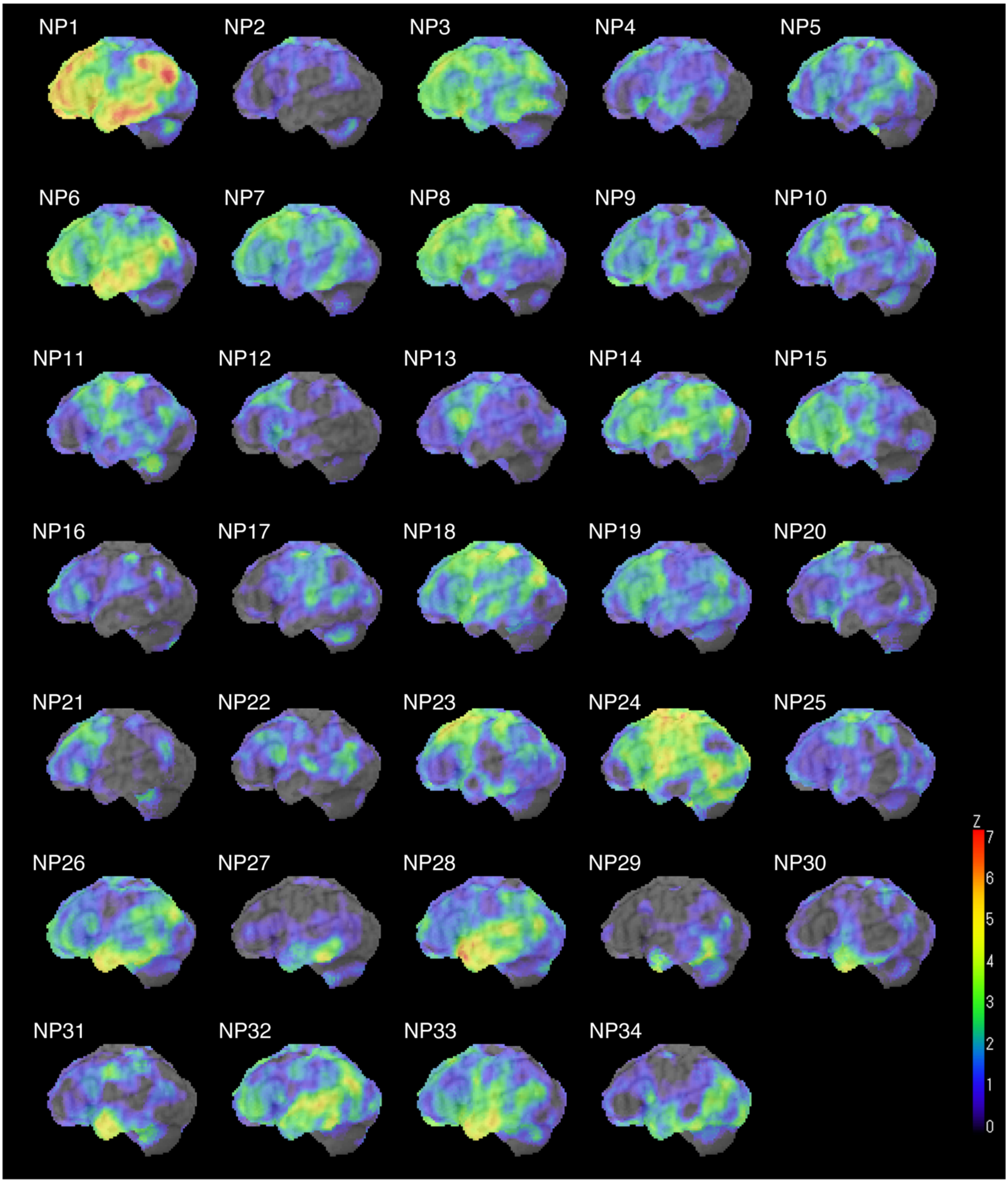

Supplementary Fig. 2. Scatter plots depicting the principal components (sPC1–3, 5) for all AD-PPA and non-AD-PPA cases. The left panel shows sPC1 versus sPC2, the middle panel displays sPC1 versus sPC3, and the right panel presents sPC1 versus sPC5. Data points are symbolized as follows: circles for nfvPPA, squares for svPPA, right-pointing triangles for lvPPA, left-pointing triangles for nf+lv, plus signs for anomic PPA, and diamonds for all other cases. AD-PPA cases are indicated in red, while non-AD-PPA cases are shown in black.

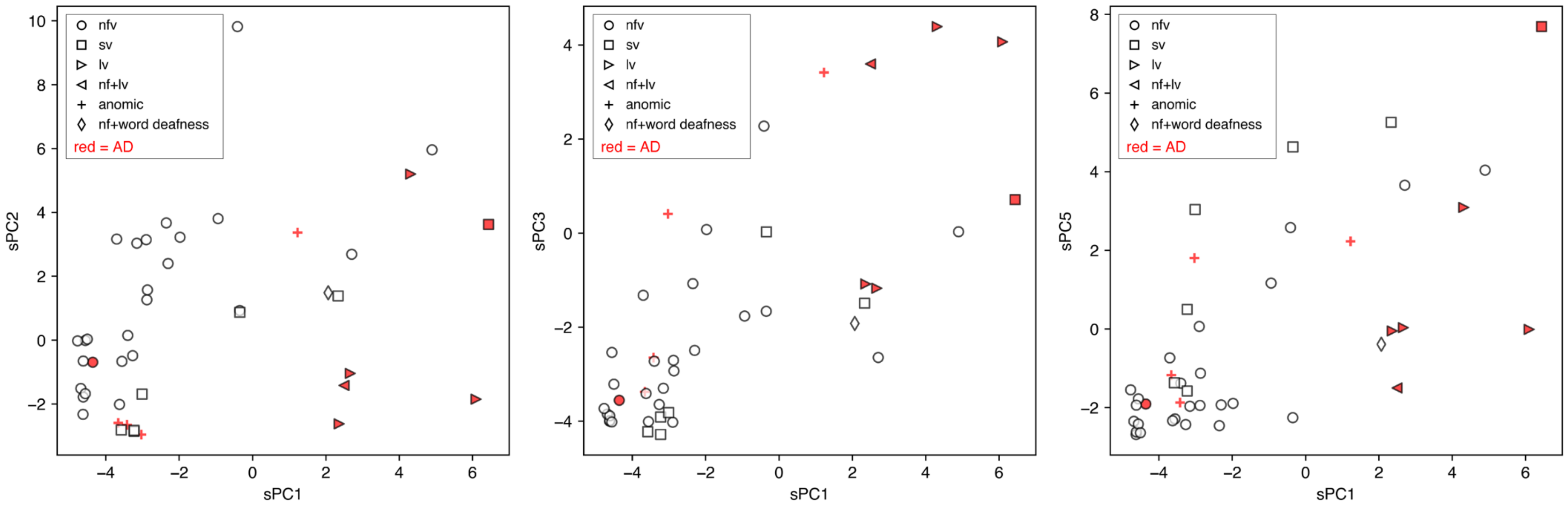

Supplement: Supplementary file 1 — Supplementary Material 1 [file 10072_2025_8100_MOESM1_ESM.pdf]
