# Supplementary material for: Distinct cerebral perfusion patterns and linguistic profiles in Alzheimer’s disease-related primary progressive aphasia
Source: Neurol Sci. 2025 Mar 24;46(7):3071–83. doi: 10.1007/s10072-025-08100-2 (PMC12152036; doi:10.1007/s10072-025-08100-2)
Supplement: Supplementary file 2 — Supplementary Material 2 [file 10072_2025_8100_MOESM2_ESM.pdf]

Supplementary Table 1. Demographics of AD-PPA group (#P1–#P11).

**Patient**	**P1**	**P2**	**P3**	**P4**	**P5**	**P6**	**P7**	**P8**	**P9**	**P10**	**P11**
**Age** [years]	72	69	57	74	78	80	75	81	76	81	73
**Disease duration** [years]	6.5	2	4.2	1.3	2.8	3.5	2.5	2.5	10	1.5	3.7
**Sex**	M	F	F	F	M	F	M	M	F	M	F
**Clinical subtype**	lv	lv	lv	lv	nfv	sv	anomic	anomic	anomic	anomic	nf+lv
**CSF**											
p-tau [pg/mL]	115	135	68.4	102	73.1	68.3	74.4	60.2	192	79.5	89.1
Aβ1-42/t-tau	0.532	0.826	0.765	0.736	1.094	1.860	1.124	1.540	1.047	1.796	0.592
**Education**	16	12	12	9	16	16	12	16	14	14	12
**CDR, overall**	0.5	0.5	1	0.5	0.5	2	1	0.5	0.5	0.5	0.5
**MMSE** (/30)	16	19	18	17	24	8	21	19	23	25	14
**RCPM** (/36)	26	15	26	20	27	21	28	27	31	27	19
**Apraxia of speech**	0	0	0	0	1	0	0	0	0	0	1
**Agrammatism**	0	0	0	0	1	0	0	0	0	0	0
**WAB**											
Aphasia Quotient (/100)	100	66.8	64.6	69.2	80.6	59.4	87.4	90.6	93.2	89.4	58.4
Spontaneous speech (/20)	14	13	16	16	13	13	17	17	19	17	10
Auditory comprehension (/10)	9.4	8.8	5.5	7.6	9.5	6.7	10	10	9.6	9.9	7.8
Repetition (/10)	8.2	6.9	4.4	6.7	9.2	9.1	9.8	9.8	10	10	6.2
Naming (/10)	5.9	4.7	6.4	4.3	8.6	0.9	6.9	8.5	8	7.8	5.2
Reading (/10)	8.4	7.5	6.4	9.6	8	4.8	9	9.6	8.4	9.7	6.8
Writing (/10)	6.9	5.2	8.4	8.1	7.2	3.6	8.1	7.6	9.9	9.5	5.5
Kanji word dictation (/6)	2.5	0	5	1.5	3	1	4	3.5	4.5	5	1
Kana word dictation (/6)	5.5	5.5	6	5.5	3.5	5	6	6	6	6	6
Praxis, left (/10)	10	9.8	8.2	9	9.8	8.5	10	10	9.2	9.8	10
Praxis, right (/10)	10	9.8	8.2	9	9.8	8.3	10	10	9.2	9.6	10
Constructional/visuospatial (/10)	8.7	6.8	8.7	7.4	8.3	6.5	8.9	6.4	9.1	8.5	5.9
Calculation (/24)	24	18	22	22	24	14	24	6	24	24	22
**Token test**											
Part I-V (/23)	23	19.5	11	19.5	23	19.5	21	22	22.5	22.5	17.5
Part VI (/13)	11	8	2	4	5	5	9	8	10	8	8
**TLPA**											
Naming, high-familiarity objects (/100)	64	56	79	61	88	28	71	75	83	69	51
Naming, low-familiarity objects (/100)	25	16	27	7	67	2	29	50	50	44	20
Auditory comprehension, high-familiarity objects (/100)	86	91	93	91	98	79	93	85	98	83	93
Auditory comprehension, low-familiarity objects (/100)	82	86	75	79	93	58	86	80	95	78	84
Naming, verb (/40)	14	22	17	18	33	6	34	28	34	37	15
Auditory comprehension, verb (/40)	37	38	34	34	40	32	39	39	39	39	38
**Span**											
Digit, forward	5	3	4	4	4	6	5	5	6	6	3
Digit, backward	3	2	3	4	3	3	4	4	3	4	2
**Letter fluency (ka)**	3	6	5	4	2	0	4	10	7	5	1
**Semantic fluency (animal)**	3	3	9	5	8	1	13	10	8	11	3

Supplementary Table 2. Demographics of non-AD-PPA group (#NP1–#NP34).

**Patient**	**NP1**	**NP2**	**NP3**	**NP4**	**NP5**	**NP6**	**NP7**	**NP8**	**NP9**	**NP10**
**Age** [years]	69	73	78	69	65	72	75	67	73	69
**Disease duration** [years]	1.5	1	1.5	1.4	0.7	1.4	0.5	1	5.3	7.4
**Sex**	M	F	M	M	M	F	F	F	M	M
**Clinical subtype**	nfv	nfv	nfv	nfv	nfv	nfv	nfv	nfv	nfv	nfv
**CSF**										
p-tau [pg/mL]	25	33.6	25	68.2	41.5	36.2	71.4	32.9	44.1	45.9
Aβ1-42/t-tau	2.906	5.371	5.488	3.842	5.314	5.952	5.302	5.219	7.893	6.222
**Education**	13	9	12	14	16	14	13	16	9	12
**CDR, overall**	0.5	0	0.5	0.5	0	0.5	0	0.5	0.5	0.5
**MMSE** (/30)	17	24	3	23	26	28	29	22	23	15
**RCPM** (/36)	27	27	11	10	31	29	27	31	33	28
**Apraxia of speech**	0	1	1	1	1	1	1	1	1	1
**Agrammatism**	1	1	1	1	0	1	1	1	0	1
**WAB**										
Aphasia Quotient (/100)	77.6	81.4	34.2	73.8	92.4	73	88.2	81	92	23.8
Spontaneous speech (/20)	16	13	4	14	18	14	17	16	18	2
Auditory comprehension (/10)	7.3	10	5	7.3	9.5	7	9.2	8	9.4	8.3
Repetition (/10)	8.8	9.2	5.1	8.3	9.6	8.4	8.7	8.6	10	0
Naming (/10)	6.7	8.5	3	7.3	9.1	7.1	9.2	7.9	8.6	1.6
Reading (/10)	7.5	7.9	3.7	6.6	9.4	7.6	8.8	8.2	9.8	6.9
Writing (/10)	7.2	7.8	2.4	8.8	10	10	10	9.6	7.8	4.8
Kanji word dictation (/6)	3	4.5	2.5	5	6	6	5.5	5	2.5	4
Kana word dictation (/6)	5.5	6	0	6	6	6	6	6	5.5	0.5
Praxis, left (/10)	8.3	9.7	5.8	8.3	10	9.7	10	9.7	9.8	8.3
Praxis, right (/10)	8.3	9.8	5.7	8.3	10	10	10	9.7	9.8	8.3
Constructional/visuospatial (/10)	8.9	8.6	2.4	6.1	9.4	8.4	8.9	9.3	9.4	8.6
Calculation (/24)	24	24	2	22	24	24	24	24	24	24
**Token test**										
Part I-V (/23)	20	20	7.5	18	23	21.5	21.5	11	21.5	16
Part VI (/13)	3	7	0	0	11	3	6	0	8	3
**TLPA**										
Naming, high-familiarity objects (/100)	76	94	43	71	100	92	97	86	93	NE
Naming, low-familiarity objects (/100)	33	86	20	31	95	51	81	56	73	NE
Auditory comprehension, high-familiarity objects (/100)	97	100	33	90	100	100	100	96	100	97
Auditory comprehension, low-familiarity objects (/100)	86	99	23	69	97	98	100	85	90	97
Naming, verb (/40)	34	34	3	20	39	16	33	31	37	NE
Auditory comprehension, verb (/40)	36	40	16	32	40	37	40	37	40	40
**Span**										
Digit, forward	5	3	3	4	4	5	4	4	4	2
Digit, backward	4	3	NE	3	3	2	4	2	4	3
**Letter fluency (ka)**	NE	3	NE	3	5	0	5	3	6	1
**Semantic fluency (animal)**	7	11	0	4	13	8	16	5	11	0

Patient	NP11	NP12	NP13	NP14	NP15	NP16	NP17	NP18	NP19	NP20
**Age** [years]	79	77	58	75	74	73	79	81	72	69
**Disease duration** [years]	3	1.8	2.9	1.7	3.7	3.2	1.9	4	0.5	2.7
**Sex**	M	M	F	M	M	M	F	M	F	F
**Clinical subtype**	nfv	nfv	nfv	nfv	nfv	nfv	nfv	nfv	nfv	nfv
**CSF**										
p-tau [pg/mL]	27.2	39.1	35.8	35.8	54.9	44.6	37.1	31.1	27.8	25
Aβ1-42/t-tau	7.610	2.746	6.503	8.880	6.279	5.449	8.402	4.981	5.528	5.328
**Education**	12	12	16	12	9	16	12	12	12	11
**CDR, overall**	0.5	1	0	0.5	0.5	0.5	0.5	1	0.5	0.5
**MMSE** (/30)	26	23	28	23	11	26	30	23	26	24
**RCPM** (/36)	22	16	33	30	22	26	30	21	23	26
**Apraxia of speech**	1	1	1	1	1	1	1	1	1	1
**Agrammatism**	1	1	1	1	1	1	0	1	1	1
**WAB**										
Aphasia Quotient (/100)	65	80.8	82.6	75.4	52.2	78.4	93.4	72	84.8	78
Spontaneous speech (/20)	8	16	14	12	12	12	18	10	15	13
Auditory comprehension (/10)	9.4	7.3	9.4	8.8	4.8	9.2	10	9.2	8.6	8.4
Repetition (/10)	7.2	8.8	8.8	9	2.8	10	9.7	8.6	10	9.4
Naming (/10)	7.9	6.3	9.1	7.9	6.5	8	9	8.2	8.8	8.2
Reading (/10)	9.4	5.8	10	9.6	6.7	9.4	10	9.9	8.4	9.7
Writing (/10)	7.5	8.3	8.7	8.7	6.2	10	10	5.4	9.9	8
Kanji word dictation (/6)	5.5	3	6	5	4	6	5	4	NE	5
Kana word dictation (/6)	6	6	6	6	5	6	6	5	NE	6
Praxis, left (/10)	7.8	9.3	9.8	9.7	6.7	9.8	10	8.7	9.7	8.3
Praxis, right (/10)	8.2	9.3	9.8	9.8	6.7	9.8	10	8.3	9.7	7.7
Constructional/visuospatial (/10)	7.1	6.8	9.4	8.6	7.2	7.5	8.5	6.7	7.8	7.5
Calculation (/24)	24	22	22	24	22	24	24	16	24	22
**Token test**										
Part I-V (/23)	22.5	NE	20	16	NE	20	22	18.5	21	19
Part VI (/13)	8	NE	12	3	NE	6	12	5	8	6
**TLPA**										
Naming, high-familiarity objects (/100)	81	84	97	83	77	83	96	89	94	85
Naming, low-familiarity objects (/100)	55	68	90	66	65	68	84	71	79	54
Auditory comprehension, high-familiarity objects (/100)	94	80	100	90	NE	99	99	96	99	99
Auditory comprehension, low-familiarity objects (/100)	90	76	99	97	NE	91	99	90	97	92
Naming, verb (/40)	21	37	31	30	35	34	40	35	37	27
Auditory comprehension, verb (/40)	39	32	40	36	NE	40	40	39	40	39
**Span**										
Digit, forward	5	4	5	3	NE	5	4	3	6	5
Digit, backward	5	3	4	2	NE	3	4	3	5	2
**Letter fluency (ka)**	4	0	5	2	0	6	5	3	3	6
**Semantic fluency (animal)**	9	6	13	5	8	9	12	6	8	7

Patient	NP21	NP22	NP23	NP24	NP25	NP26	NP27	NP28	NP29	NP30	NP31
**Age** [years]	79	70	72	69	76	70	59	78	48	63	76
**Disease duration** [years]	3	3.3	1.8	6	2	4	4.5	2.3	5.5	3.8	3
**Sex**	F	M	M	M	F	M	F	M	M	F	M
**Clinical subtype**	nfv	nfv	nfv	nfv	nfv	sv	sv	sv	sv	sv	sv
**CSF**											
p-tau [pg/mL]	32	25	50.4	26.7	41.5	55.2	56.6	26.4	29.2	33.8	35.1
Aβ1-42/t-tau	3.817	5.750	5.575	5.638	5.122	2.402	4.475	4.990	4.897	6.981	5.298
**Education**	12	15	9	12	9	16	12	16	16	16	21
**CDR, overall**	0.5	0	0.5	1	0.5	0.5	0.5	2	0.5	0	0.5
**MMSE** (/30)	17	26	20	17	25	24	20	18	16	30	28
**RCPM** (/36)	26	22	19	21	29	29	33	30	34	34	34
**Apraxia of speech**	1	1	1	1	1	0	0	0	0	0	0
**Agrammatism**	1	1	1	1	1	0	0	0	0	0	0
**WAB**											
Aphasia Quotient (/100)	73.8	77.2	73	58	80.6	90.4	59.8	68.6	57	88.4	83.6
Spontaneous speech (/20)	12	12	15	7	18	19	14	14	14	16	17
Auditory comprehension (/10)	8.2	8	7.4	6.4	9	9.9	6.5	9	4.9	9.8	9.2
Repetition (/10)	9.2	9.6	8.2	8.6	9	9.8	8.2	9.9	9	10	10
Naming (/10)	7.5	9	5.9	7	8.5	6.5	1.2	1.4	0.6	8.4	5.6
Reading (/10)	7.5	9.8	9.8	5.8	9.9	10	6.1	4.3	4.4	9.8	10
Writing (/10)	7.8	9.6	8	6.4	8.7	10	6.4	4.9	6.7	10	9.8
Kanji word dictation (/6)	5	5	5	2	5	4.5	2.5	1.5	0	6	5
Kana word dictation (/6)	6	6	5.5	4.5	6	6	6	6	6	6	6
Praxis, left (/10)	9.3	10	9.2	9.2	9.7	9.7	7.5	7	5.8	9.7	9.8
Praxis, right (/10)	9.2	10	9.2	9.7	9.7	9.7	7.5	7	5.8	9.7	9.8
Constructional/visuospatial (/10)	7.4	7.7	6.2	6.4	8.8	9	9.4	8.4	8.7	9.5	9.7
Calculation (/24)	20	24	16	20	24	24	24	24	22	24	24
**Token test**											
Part I-V (/23)	16.5	18.5	17.5	NE	22.5	23	23	18.5	NE	23	23
Part VI (/13)	1.5	7	1	NE	10	11	5	3	NE	13	11
**TLPA**											
Naming, high-familiarity objects (/100)	87	92	71	75	97	62	36	27	13	90	74
Naming, low-familiarity objects (/100)	67	72	41	46	74	23	6	3	1	39	20
Auditory comprehension, high-familiarity objects (/100)	84	100	91	83	100	83	53	55	17	99	98
Auditory comprehension, low-familiarity objects (/100)	81	97	81	77	98	70	21	30	5	92	87
Naming, verb (/40)	28	36	23	22	33	NE	29	20	7	31	29
Auditory comprehension, verb (/40)	34	40	33	36	39	NE	33	35	10	38	39
**Span**											
Digit, forward	4	4	4	4	5	5	4	6	6	5	6
Digit, backward	3	3	4	2	4	5	2	4	4	3	5
**Letter fluency (ka)**	3	3	0	3	0	11	1	0	3	12	11
**Semantic fluency (animal)**	5	12	5	5	14	8	0	2	0	14	7

Patient	NP32	NP33	NP34
**Age** [years]	64	72	82
**Disease duration** [years]	3.7	2.5	1
**Sex**	M	M	M
**Clinical subtype**	nf + word deafness	anomic	anomic
**CSF**			
p-tau [pg/mL]	34.7	35.3	42.3
Aβ1-42/t-tau	8.325	3.153	1.632
**Education**	12	12	12
**CDR, overall**	0.5	0.5	0.5
**MMSE** (/30)	29	28	25
**RCPM** (/36)	33	32	26
**Apraxia of speech**	1	0	0
**Agrammatism**	1	0	0
**WAB**			
Aphasia Quotient (/100)	66.4	76.8	90
Spontaneous speech (/20)	13	13	18
Auditory comprehension (/10)	7.3	9.6	9.6
Repetition (/10)	5.2	8.3	9.5
Naming (/10)	7.7	7.5	7.9
Reading (/10)	8.2	9.6	8
Writing (/10)	7.8	9.6	10
Kanji word dictation (/6)	6	NE	5.5
Kana word dictation (/6)	5.5	NE	6
Praxis, left (/10)	9.3	9.8	9
Praxis, right (/10)	9.3	10	9
Constructional/visuospatial (/10)	9.4	9.4	8.6
Calculation (/24)	24	24	8.6
**Token test**			
Part I-V (/23)	15.5	22	21.5
Part VI (/13)	4.5	11	11
**TLPA**			
Naming, high-familiarity objects (/100)	83	75	86
Naming, low-familiarity objects (/100)	44	32	37
Auditory comprehension, high-familiarity objects (/100)	95	97	92
Auditory comprehension, low-familiarity objects (/100)	95	88	89
Naming, verb (/40)	23	29	32
Auditory comprehension, verb (/40)	38	40	33
**Span**			
Digit, forward	4	5	7
Digit, backward	5	4	6
**Letter fluency (ka)**	5	5	15
**Semantic fluency (animal)**	11	5	10

Supplementary Table 3. sPCA results for SPECT perfusion patterns in left hemisphere ROIs.

Region of Interest	sPC1	sPC2	sPC3	sPC4	sPC5
**Frontal**					
Superior frontal, L	0	0.2678	0	0	0
Middle frontal, L	0	0.3215	0	0	0
Inferior frontal, L	0	0.2658	0	0	0.0980
Medial frontal, L	0	0.2486	0	0	0
Paracentral lobule, L	0	0.1196	0.1065	0	0
Subcallosal, L	0.0080	0.1561	0	0	0.2207
Precentral, L	0	0.2760	0	0	0
**Parietal**					
Postcentral, L	0	0.1252	0.1444	0.0186	0
Superior parietal, L	0	0	0.4762	0	0
Inferior parietal, L	0.2427	0	0.2039	0.0688	0
Supramarginal, L	0.3811	0	0.0891	0	0
Angular, L	0.2025	0	0.2669	0.0766	0
Precuneus, L	0	0	0.3527	0	0
**Temporal**					
BA42 (Transverse temporal), L	0.2501	0	0.1415	0	0.0290
BA22 (Superior temporal), L	0.4131	0	0.0016	0	0
BA21 (Middle temporal), L	0.5397	0	0	0	0
BA20 (Inferior temporal), L	0.3073	0	0	0	0.2543
BA38 (Temporal pole), L	0.0176	0	0.0166	0	0.5041
**Basal**					
Lingual, L	0	0	0	0	0
Fusiform, L	0.2101	0	0	0	0.0533
Parahippocampal, L	0.0331	0	0	0	0.5113
**Occipital**					
Superior occipital, L	0	0	0.5701	0	0
Middle occipital, L	0.0074	0	0.2507	0	0.0202
Inferior occipital, L	0	0	0.0001	0	0
Cuneus, L	0	0	0.0799	0	0
**Medial**					
Cingulate, L	0	0.2039	0.0718	0	0
Anterior cingulate, L	0	0.1810	0	0	0.2676
Posterior cingulate, L	0	0	0.2603	0	0.0557

Supplementary Table 4. sPCA results for SPECT perfusion patterns in right hemisphere ROIs.

Region of Interest	sPC1	sPC2	sPC3	sPC4	sPC5
**Frontal**					
Superior frontal, R	0	0.1406	0	0.1014	0
Middle frontal, R	0	0.1915	0	0.1140	0
Inferior frontal, R	0	0.2289	0	0.0238	0.0739
Medial frontal, R	0	0.1689	0	0.0328	0
Paracentral lobule, R	0	0.1107	0.0101	0	0
Subcallosal, R	0	0.0230	0.0008	0	0.1022
Precentral, R	0	0.1599	0	0	0
**Parietal**					
Postcentral, R	0	0.1843	0.0063	0	0
Superior parietal, R	0	0.2580	0	0.0265	0
Inferior parietal, R	0	0	0	0.4493	0
Supramarginal, R	0	0	0	0.6386	0
Angular, R	0	0.0352	0	0.4595	0
Precuneus, R	0	0.1952	0	0.0695	0
**Temporal**					
BA42 (Transverse temporal), R	0.1769	0.1747	0.0399	0	0.0223
BA22 (Superior temporal), R	0.1932	0	0.0187	0.0961	0
BA21 (Middle temporal), R	0.1471	0	0	0.2719	0.0067
BA20 (Inferior temporal), R	0	0	0	0.2284	0.1729
BA38 (Temporal pole), R	0	0	0	0	0.3526
**Basal**					
Lingual, R	0	0	0	0	0
Fusiform, R	0	0	0	0.0227	0.0181
Parahippocampal, R	0	0	0	0.0269	0.2377
**Occipital**					
Superior occipital, R	0	0.1615	0.0255	0.0341	0
Middle occipital, R	0	0.0286	0.0217	0	0
Inferior occipital, R	0	0	0	0	0
Cuneus, R	0	0	0	0	0
**Medial**					
Cingulate, R	0	0.2020	0.0731	0	0
Anterior cingulate, R	0	0.1524	0	0	0.2350
Posterior cingulate, R	0	0.2251	0	0	0

Supplementary Table 5. SPECT perfusion patterns projected onto sPCs for each participant in non-AD-PPA group.

Patient	Subtype	sPC1	sPC2	sPC3	sPC4	sPC5
**NP1**	nfv	4.898	5.957	0.027	-2.033	4.036
**NP2**	nfv	-4.619	-2.326	-4.001	-3.576	-2.686
**NP3**	nfv	-0.937	3.804	-1.766	-0.094	1.165
**NP4**	nfv	-3.559	-0.668	-4.010	-3.885	-2.288
**NP5**	nfv	-2.877	1.262	-2.707	0.097	-1.947
**NP6**	nfv	2.707	2.687	-2.647	0.624	3.652
**NP7**	nfv	-2.300	2.398	-2.496	0.551	-1.932
**NP8**	nfv	-2.349	3.668	-1.076	-0.766	-2.461
**NP9**	nfv	-2.863	1.569	-2.932	1.079	-1.128
**NP10**	nfv	-4.556	-0.013	-2.540	-2.750	-1.779
**NP11**	nfv	-3.152	3.034	-3.302	-0.069	-1.967
**NP12**	nfv	-4.680	-1.516	-3.852	-3.463	-2.350
**NP13**	nfv	-4.613	-1.783	-3.990	-3.795	-2.620
**NP14**	nfv	-0.346	0.925	-1.665	-2.582	-2.258
**NP15**	nfv	-2.897	3.143	-4.024	-1.454	0.062
**NP16**	nfv	-4.613	-0.656	-3.887	-1.452	-1.939
**NP17**	nfv	-3.624	-2.016	-3.413	-3.842	-2.336
**NP18**	nfv	-1.975	3.221	0.074	0.528	-1.894
**NP19**	nfv	-3.400	0.146	-2.727	-2.607	-1.381
**NP20**	nfv	-4.768	-0.024	-3.732	-3.885	-1.550
**NP21**	nfv	-4.558	-1.679	-4.018	-3.787	-2.420
**NP22**	nfv	-3.266	-0.488	-3.647	0.322	-2.432
**NP23**	nfv	-3.701	3.162	-1.324	0.292	-0.736
**NP24**	nfv	-0.409	9.817	2.274	-1.423	2.576
**NP25**	nfv	-4.502	0.029	-3.215	-3.232	-2.641
**NP26**	sv	-0.344	0.871	0.026	-0.318	4.627
**NP27**	sv	-3.226	-2.857	-4.286	-3.521	-1.580
**NP28**	sv	2.335	1.379	-1.490	1.284	5.251
**NP29**	sv	-3.579	-2.815	-4.225	-3.862	-1.373
**NP30**	sv	-3.225	-2.831	-3.913	-3.556	0.498
**NP31**	sv	-3.009	-1.693	-3.817	-4.090	3.034
**NP32**	nf+word deafness	2.062	1.482	-1.921	0.522	-0.386
**NP33**	anomic	0.278	0.582	-4.176	-3.874	4.543
**NP34**	anomic	-2.249	-2.094	-2.787	-3.722	1.556
